# Effects of Spirulina on Mixing, Rheology, and Structure in Wheat Dough

**DOI:** 10.3390/foods15091603

**Published:** 2026-05-06

**Authors:** Miao He, Huizhen Chen, Yingguo Lyu, Chenchen Dong, Xueqin Li, Kunlun Liu

**Affiliations:** 1 College of Food Science and Engineering, Henan University of Technology, Zhengzhou 450001, China; he15037336263@163.com (M.H.); chenchendong1023@163.com (C.D.); xueqin1216@sina.cn (X.L.); knlnliu@126.com (K.L.); 2 Henan Province Wheat-Flour Staple Food Engineering Technology Research Centre, Zhengzhou 450001, China

**Keywords:** Spirulina powder, dough rheology, gluten network, protein aggregation, water distribution

## Abstract

Spirulina, a nutrient-rich microalga, was incorporated into wheat flour at 0–4% (flour basis) to evaluate its effects on dough mixing, rheology, water distribution, microstructure, and protein aggregation. The water absorption value increased from 56.2% (control) to 58.8–60.4% with Spirulina addition; however, the change was not statistically significant (*p* > 0.05). At 1–3% addition, development time increased from 2.2 to 2.4–2.7 min and stability from 4.9 to 6.4 min, while 4% addition reduced stability and increased weakening. Rheological measurements showed that G′ and G″ decreased by 23–33%, while tan δ increased from 0.37 to 0.38–0.39, indicating reduced viscoelastic strength. The LF-NMR results showed that A_21_ exhibited an increasing trend (*p* > 0.05), accompanied by a prolongation of T_22_, indicating enhanced water mobility and reduced binding strength. Microscopy showed a more continuous gluten network at 1–3%, whereas discontinuities appeared at 4%. SDS-PAGE indicated increased >60 kDa aggregates at 3% but reduced intensity at 4%. Overall, 1–3% Spirulina, particularly 3%, was associated with improved mixing tolerance and more uniform structural characteristics.

## 1. Introduction

During the kneading process of wheat flour and water, gluten proteins hydrate and expand to form a continuous three-dimensional network. This viscoelastic dough is foundational for the production of various food items. Dough rheology is a critical determinant of both processing behavior and final product quality [1]. Multiple factors—such as flour type, ingredient formulation, and processing techniques—interact with gluten proteins and moisture content to influence dough structure and functionality [2,3,4,5,6,7,8,9,10]. Optimizing these parameters allows for the tailored production of specific foods, like noodles and steamed bread [11]. Consequently, an in-depth understanding of dough rheological properties is essential for advancing cereal processing and preparation technologies.

Spirulina, a nutrient-dense aquatic microalga, is rich in bioactive compounds. It contains 50% to 70% high-quality plant protein and features an optimal amino acid profile, including all nine essential amino acids for adults [12]. Beyond protein, it provides essential vitamins, algal polysaccharides, minerals, and unsaturated fatty acids (such as γ-linolenic acid), offering a comprehensive nutritional profile. Spirulina also exhibits functional health benefits, including immunomodulatory, antioxidant, hypoglycemic, and hypolipidemic effects [13]. Incorporating Spirulina into food aligns with modern consumer demands for sustainable, health-promoting functional foods.

In recent years, Spirulina has attracted growing attention as an ingredient in certified health supplements and functional foods, such as cookies, meal replacements, and tofu [14,15,16]. Its incorporation into staple foods is regarded as a promising approach to enhance nutritional value. Indeed, Lames et al. [17] demonstrated that Spirulina fortification significantly improved the protein quality of macaroni, while Montevecchi et al. [18] reported that Spirulina altered dough rheology through interactions with the dough matrix, increasing water absorption and toughness.

Nevertheless, current studies on Spirulina in wheat-based systems have largely focused on nutritional fortification of products such as bread and cookies [19], the effects of fresh Spirulina microcapsules on noodle quality [20], and the molecular regulation of gluten aggregation by different levels of whole Spirulina biomass [21]. In general, these investigations have mainly addressed physicochemical characteristics and gluten protein structural changes, whereas the impact of Spirulina on dough rheological properties and microstructural evolution remains insufficiently understood. This knowledge gap is especially important in wheat flour-based systems, where dough development behavior plays a crucial role in determining final product quality.

To bridge this gap, this study systematically investigates the impact of Spirulina powder on the rheological properties and underlying mechanisms of wheat dough. Building on our prior work [22], which showed that Spirulina above 6% weakens dough, the present study focuses on a refined addition range (0–4%) and, for the first time, investigates the dynamic evolution of dough properties across three distinct mixing stages (formation, stabilization, and weakening). Specifically, we examine the effects of varying Spirulina concentrations and kneading durations on dough mixing characteristics, dynamic rheological behavior, moisture distribution, microstructural changes, and protein molecular weight. This study aims to elucidate the interactions between Spirulina and the dough matrix, providing a theoretical basis for its future application in wheat flour-based food products and offering innovative perspectives for the functional staple food industry.

## 2. Materials and Methods

### 2.1. Materials

Xiangmanyuan Special Grade One Wheat Flour, characterized by a protein content of 11.2%, wet gluten content of 38.4%, and ash content of 0.56%, was procured from Yihai Kerry Food Industry Co., Ltd. (Shanghai, China). Spirulina (protein approximately 65%, lipids approximately 7%, ash approximately 6%, and moisture approximately 5%. The particle size description has been revised to “D90 < 50 μm” and the color is dark green (L* = 25.3, a* = −8.2, b* = 12.5)) was obtained from Etuoke Banner Derong Algae Industry Co., Ltd. (Ordos, China). Tris, glycine, bromophenol acrylamide, methylene diacrylamide, ammonium persulfate, and glycerol were sourced from Tianjin Comiao Chemical Reagent Co., Ltd. (Tianjin, China). SDS was acquired from Tianjin Kaitong Chemical Reagent Co., Ltd. (Tianjin, China). Komas Bright Blue was obtained from Shanghai ASUS Fine Chemicals Co., Ltd. (Shanghai, China). Anhydrous ethanol and glacial acetic acid were sourced from Tianjin Zhiyuan Chemical Reagent Co., Ltd. (Tianjin, China). Concentrated hydrochloric acid was procured from Tianjin Fengchuan Chemical Reagent Co., Ltd. (Tianjin, China). β-mercaptoethanol was purchased from Kondis Chemical Co., Ltd. (Wuhan, China). TEMED and Bright Green were obtained from Shanghai Maclean Biochemical Technology Co., Ltd. (Shanghai, China). Low-molecular-weight protein markers were sourced from Beijing Solabao Technology Co., Ltd. (Beijing, China). Iodine and potassium iodide were purchased from Shandong Baiqianhua Co., Ltd. (Linyi, China). All chemical reagents obtained were of analytical grade.

### 2.2. Preparation of Mixed Flour and Doughs

For each batch, 200 g of flour (14% moisture basis) was used. Spirulina powder was added at 0–4% (*w*/*w*) as a replacement for flour weight. The water addition for each formulation is as follows: control (112.4 mL), 1% (117.6 mL), 2% (119.2 mL), 3% (117.72 mL), and 4% (120.8 mL). These values were determined using a farinograph to achieve a constant consistency of 500 FU, as is standard in dough rheology studies (AACC Method 54-21) to ensures that comparisons are made at equivalent functional stages. Mixing was carried out at 60 rpm; the dough temperature was controlled at 25 ± 1 °C. All dynamic rheology measurements were performed immediately after mixing (within 2 min, no resting), while LF-NMR, microstructure, and SDS-PAGE analyses were performed after a 10 min resting period to allow thermal and moisture equilibration.

The farinographic properties of the dough were evaluated according to AACC 54-21 standard [23]. A Brabender Farinograph (Farinograph-AT, Brabender GmbH & Co. KG, Duisburg, Germany) equipped with a 50 g mixing bowl was used. The sample mass was corrected to 50 g on a 14% moisture basis, and all measurements were performed in triplicate. The parameters determined included water absorption, development time, stability time, degree of softening, and quality number.

### 2.3. Determination of Kneading Characteristics

Flour kneading parameters were determined according to AACC 54-40A standard method [24]. A 10 g sample (14% moisture basis) was placed into the mixing bowl of a mixograph (Model 505SS, National Company, Austin, TX, USA), and the water addition was set at 85% of the farinograph water absorption. The mixing speed was 88 rpm, and measurements were performed in triplicate. After securing the bowl, the mixing curve was recorded. Kneading parameters were subsequently calculated using the Mixsmart program, including peak time, 8 min bandwidth, peak curve area, and right-hand slope.

### 2.4. Determination of Dynamic Rheological Properties

A 5 g dough sample was placed on a parallel plate rheometer (DHR-1, TA Co., Westlake, OH, USA) and trimmed to an appropriate weight for measurement. The plates had a diameter of 25 mm, and the gap between the two plates was maintained at 2 mm. Any excess sample was trimmed from the edges, and silicone oil was applied around the exposed edges to prevent moisture evaporation and minimize experimental errors. Frequency scanning tests were performed at a strain of 0.1% (strain sweep tests confirmed that 0.1% strain was within the linear viscoelastic region for all samples (0–4% Spirulina)), a temperature of 25 °C, and a frequency range of 0.1 to 20 Hz [25]. The storage modulus (G′) and loss modulus (G″) of the samples as functions of frequency were recorded. The loss tangent (tan δ) was calculated by dividing G″ by G′ [26].

### 2.5. Determination of Moisture Distribution and Moisture Migration

Transverse relaxation measurements were performed using a low-field nuclear magnetic resonance analyzer (LF-NMR; MicroMR-CL-I, Niumag Electronic Technology Co., Ltd., Suzhou, China). A 3 g dough sample collected at each sampling time point was placed in a test tube with a diameter of 10 mm for measurement. The temperature was maintained at 32 ± 1 °C, the equilibration time was 10 min, and each measurement was performed in triplicate. The operating parameters were set as follows: sampling frequency (SW), 333.33 kHz; number of sampling points (TD), 133,332; waiting time (TW), 1500 ms; number of echoes (NECH), 4000; echo time (TE), 0.1 ms; and number of scans (NS), 16. To evaluate moisture distribution and migration, dough samples were collected at three defined mixing stages: Formation/Development stage: Sampling was performed at the time to reach peak consistency (development time), as defined by AACC Method 54-21. Stabilization stage: Sampling was performed at the midpoint of the stability time window (i.e., the time at which consistency remained at 500 ± 20 FU for at least 2 min). Weakening stage: Sampling was performed when the consistency decreased by 30 FU from the peak value (weakening onset).

### 2.6. Observation of Dough Microstructure

The microstructure of the dough was observed according to a modified method described by Li et al. [22]. Dough samples prepared as described in Section 2.2 were frozen at −40 °C for 2 h. The frozen samples were then sliced into thin sections (approximately 15 μm thick) using a sharp blade and mounted onto glass slides. Three biological replicates were prepared for each sample, and five fields of view were captured per replicate. Two staining approaches were used for comparison: single staining samples were stained only for starch using Lugol’s iodine solution for 1 min. In these images, starch granules appear dark blue/purple, while the gluten network remains unstained (visible as a light/transparent matrix), and Spirulina particles appear as distinct green granules. Sequential double staining samples were first stained with 0.1% (*w*/*v*) Brilliant Green for 1 min (which stains both protein and Spirulina green), followed by Lugol’s iodine solution for an additional 1 min (which stains starch dark blue/purple). After placing a coverslip, the samples were observed and photographed using an optical microscope (E5, Ningbo Shunyu Instrument Co., Ltd., Ningbo, China) at a magnification of 400×.

### 2.7. SDS–Polyacrylamide Gel Electrophoresis (SDS-PAGE)

SDS-PAGE was performed according to the method described by Yang et al. [27], with slight modifications. Protein composition was analyzed using sodium dodecyl sulfate–polyacrylamide gel electrophoresis (SDS-PAGE). Freeze-dried wheat dough and spirulina dough samples were collected from three different sampling points. For each sample, 50 mg was weighed and mixed with 1 mL of sample buffer (125 mM Tris–HCl, pH 6.8; 2% (*w*/*v*) SDS; 10% (*v*/*v*) glycerol; 5% (*v*/*v*) β-mercaptoethanol; 0.01% (*w*/*v*) bromophenol blue). The mixture was homogenized and then boiled for 5 min. Subsequently, samples were centrifuged at 12,000 rpm for 10 min, and the supernatant was collected for further analysis. Electrophoresis was performed under reducing conditions, with 5% β-mercaptoethanol included in the sample buffer. A 5% stacking gel (3.4 mL distilled water, 0.83 mL 30% acrylamide/bis-acrylamide, 0.63 mL 1.0 M Tris–HCl (pH 6.8), 50 μL 10% SDS, 75 μL 10% ammonium persulfate, and 7.5 μL TEMED) and a 12% resolving gel (3.3 mL distilled water, 4.0 mL 30% acrylamide/bis-acrylamide (29:1), 2.5 mL 1.5 M Tris–HCl (pH 8.8), 100 μL 10% SDS, 100 μL 10% ammonium persulfate, and 10 μL TEMED) were prepared. A total of 10 μg of protein was loaded per lane (1.0 μg/μL), as determined using a BCA protein assay kit with bovine serum albumin as the standard, measuring absorbance at 562 nm to ensure consistent loading. Electrophoresis was performed under constant conditions of 100 V and 20 mA until the tracking dye reached the bottom of the gel. After electrophoresis, the gels were stained with Coomassie Brilliant Blue for 2 h and then destained. The destained gels were imaged and analyzed using Image J (1.54 n) software (Bio-Rad, Hercules, CA, USA). Densitometric analysis was performed by normalizing the total intensity of each lane. All experiments were conducted in triplicate.

### 2.8. Statistical Analysis

Statistical analysis was performed using SPSS 19.0 software (IBM, Armonk, NY, USA). Results were expressed as mean ± standard deviation. Significant differences between samples were determined using analysis of variance (ANOVA), and different lowercase letters indicate significant differences at *p* < 0.05. All experiments were performed in triplicate (*n* = 3). Figures were generated using Origin 2021 software.

## 3. Results

### 3.1. Comparison of Flour Characteristics and Dough Mixing Properties of Wheat Flour and Spirulina Blends

**Figure 1:**
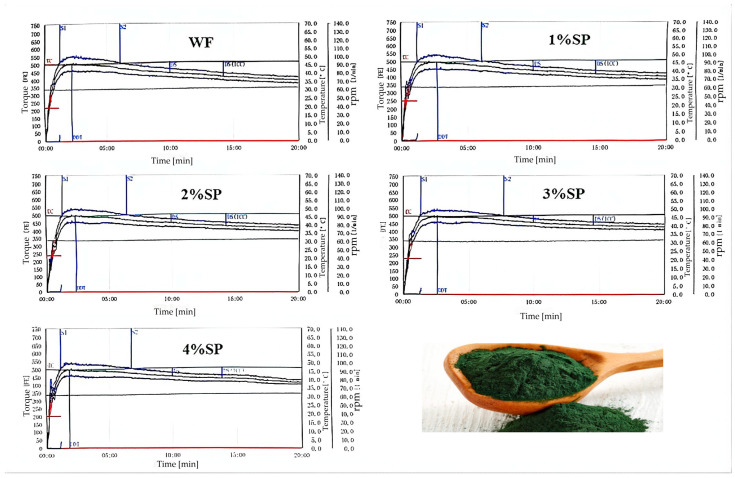
Farinograph curve of wheat flour and spirulina blends. WF: wheat flour; SP: Spirulina powder. Note: 1–4% SP indicate blends with corresponding Spirulina levels.

**Figure 2:**
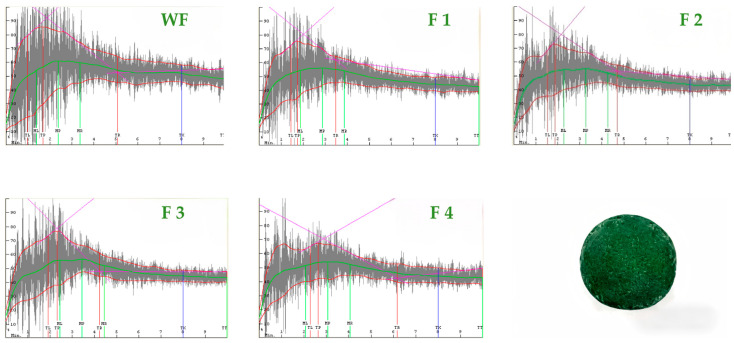
Mixograph curve of wheat flour and spirulina blends. WF: wheat flour; SP: Spirulina powder. Note: 1–4% SP indicate blends with corresponding Spirulina levels.

Farinograph and mixograph parameters are closely related to dough processing performance and product quality [28,29,30]. As shown in Figure 1 and Figure 2 and Table 1, Spirulina addition significantly affected flour characteristics and mixing behavior.

The water absorption value increased from 56.2% (control) to 58.8–60.4% with Spirulina addition; however, the change was not statistically significant (*p* > 0.05). Formation time remained relatively long when the Spirulina addition ranged from 1% to 3%. Both development time and peak time showed a trend of initial increase followed by a decrease, reaching their maximum values at 2%. In contrast, stability time and the farinograph quality number exhibited a decrease–increase pattern, whereas the 8 min bandwidth remained consistently lower than that of the control at all Spirulina addition levels. In addition, the peak curve area increased progressively as the Spirulina level increased. The weakening degree decreased at low to moderate addition levels (1–3%) but increased again at 4%. Among all samples, the dough containing 3% Spirulina exhibited the longest stability time, the highest farinograph quality number, and the lowest weakening degree, indicating that this level was the most favorable for improving dough mixing tolerance and overall stability.

From a mechanistic perspective, the increase in water absorption may be related to the presence of dietary fiber and polysaccharides in Spirulina, which contain hydrophilic groups capable of binding water [31]. The changes in development time and peak time may be associated with competition for water between Spirulina components and gluten proteins, affecting gluten hydration and network formation [32]. The reduction in stability at low addition levels may be related to gluten dilution and interference from non-gluten components, whereas the partial recovery at moderate levels may be associated with the protein contribution of Spirulina [33]. The increase in peak curve area may reflect higher mixing resistance, possibly due to the swelling and water-binding behavior of polysaccharides. The increase in weakening degree at 4% suggests that excessive addition may disrupt the dough structure [34].

Overall, the results indicate that moderate Spirulina addition (1–3%), particularly at 3%, is associated with improved mixing performance, whereas excessive addition (4%) shows an opposite trend.

### 3.2. Comparison of Dynamic Rheological Properties of Wheat Dough and Spirulina Dough

**Figure 3:**
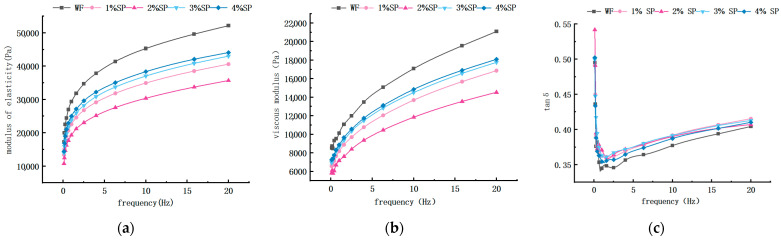
Dynamic rheological properties of wheat flour and Spirulina blends. (**a**) Storage modulus (G′); (**b**) loss modulus (G″); (**c**) loss tangent (tan δ). Notes: All experiments were conducted in triplicate (n = 3). Statistical significance was defined as *p* < 0.05, and different letters indicate significant differences among samples.

Dynamic rheological analysis showed that G′ and G″ increased with oscillation frequency, indicating typical viscoelastic behavior [35,36,37]. As shown in Figure 3, all samples exhibited tan δ < 1, suggesting elasticity-dominant behavior. With increasing Spirulina addition, G′ and G″ first decreased (1–2%) and then showed a partial increase at higher levels. The initial decrease indicates a reduction in the elastic network strength, which may be associated with gluten dilution and the presence of non-gluten components such as dietary fiber and polysaccharides that interfere with gluten network formation [38,39,40,41]. At higher addition levels, the partial recovery of G′ and G″ may be related to the physical effects of Spirulina components, such as water absorption, swelling, and filling within the dough matrix. However, the overall G′ and G″ values remained lower than those of the wheat dough control, indicating that Spirulina incorporation did not enhance the intrinsic viscoelastic strength of the gluten network. The increase in tan δ further suggests a shift toward more viscous behavior.

It should be noted that these rheological results reflect the linear viscoelastic properties of the dough under small deformation, whereas farinograph and mixograph parameters describe mixing tolerance and dough development behavior under large deformation. Therefore, the improved stability and mixing performance observed at moderate Spirulina levels (e.g., 3%) do not necessarily correspond to an increase in elastic modulus. Instead, Spirulina addition may enhance water retention and mixing resistance without strengthening the gluten network itself. This difference highlights that improvements in processing performance and reductions in linear viscoelastic strength are not contradictory but reflect distinct aspects of dough behavior. Specifically, we now propose that while Spirulina addition reduces the overall viscoelastic moduli—indicating a weaker dough structure—this does not necessarily imply poorer processing quality. It is hypothesized that the observed changes in dough rheological properties and water distribution may influence processability, mixing energy requirements, and the quality attributes of the final product; however, these assumptions require validation through direct experimental evidence in future studies.

### 3.3. Moisture Distribution and Migration

**Figure 4:**
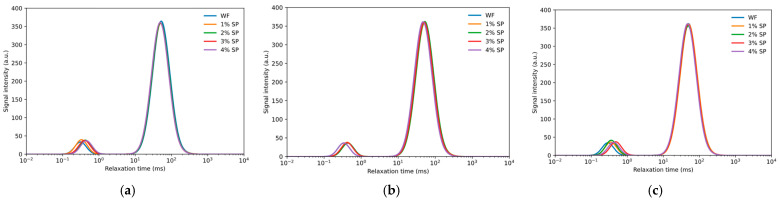
Changes in relaxation times of doughs with different Spirulina additions during mixing. (**a**) Development stage; (**b**) stabilization stage; (**c**) weakening stage.

The low-field nuclear magnetic resonance (LF-NMR) analysis shown in Figure 4 and Table 2 indicates that spirulina has an impact on the distribution and migration of water during the dough mixing process. The relaxation times T_21_, T_22_, and T_23_ correspond to strongly bound water, weakly bound water, and free water, respectively, while the peak area fractions A_21_, A_22_, and A_23_ represent their relative proportions. Since these two groups of parameters reflect different physical meanings, they were interpreted separately.

In terms of water distribution, the area fractions A_21_, A_22_, and A_23_ did not show a fully consistent pattern after Spirulina addition. Although A_21_ tended to increase in some cases, these changes were not consistently significant across all treatments. Therefore, the data do not support a strong conclusion that Spirulina increased the proportion of strongly bound water; rather, they suggest that Spirulina may have induced a redistribution of water populations within the dough matrix.

In terms of water mobility, the relaxation times provided clearer evidence of Spirulina-induced changes. In wheat dough, water progressively interacts with starch and gluten during mixing, whereas water mobility increases at later stages as the gluten network weakens [42,43]. In Spirulina-containing dough, the prolongation of T_22_ in some treatments indicated reduced binding strength and enhanced mobility of weakly bound water. This behavior suggests that Spirulina altered water–matrix interactions and weakened the restraining effect of the gluten network on water molecules. These results are consistent with the rheological data, which showed reduced viscoelasticity and structural stability after Spirulina addition.

At 1–2% Spirulina, the overall water migration pattern remained similar to that of the control dough, whereas more pronounced changes were observed at 3–4%, likely due to the combined effects of gluten dilution and the participation of Spirulina components in water redistribution [44]. Overall, Spirulina appeared to influence water mobility more clearly than water population distribution, indicating that its main effect was to modify the interaction between water and the dough matrix rather than to consistently increase the proportion of tightly bound water.

### 3.4. Effect of Different Spirulina Additions on Dough Microstructure During Mixing

**Figure 5:**
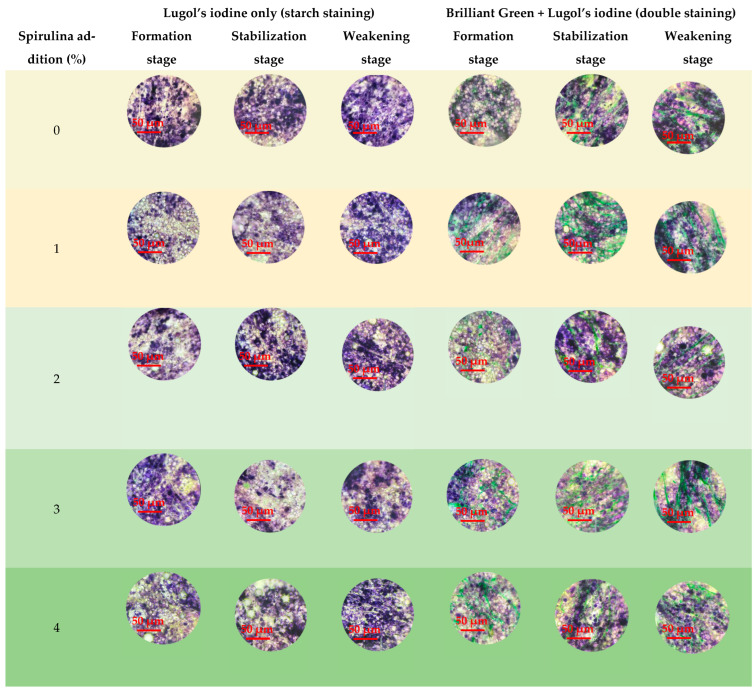
Microstructure of doughs with different Spirulina additions during mixing. **Notes:** Microscope magnification: 40× objective lens and 10× eyepiece lens (total magnification 400×).

Microscopic observations revealed clear changes in dough microstructure with Spirulina addition during mixing (Figure 5). In samples stained only for starch, the number of purple-stained starch granules decreased gradually as the Spirulina level increased, while light green Spirulina particles became more abundant. In samples co-stained for starch and protein, Spirulina particles appear as discontinuous, dark green granular structures dispersed within the dough matrix, while the gluten protein network forms a continuous, interconnected fibrous or sheet-like structure with a lighter yellowish-green appearance.

At the dough development stage, the gluten network in the wheat flour control appeared discontinuous, indicating that the network structure was not yet fully developed. With Spirulina addition, particularly at low to moderate levels, the gluten network appeared more continuous and more regularly distributed, changing from a discontinuous knot-like appearance to a more elongated strip-like structure.

At the stabilization stage, the gluten network became clearer and denser as the Spirulina addition increased up to 3%. Among all samples, the 3% Spirulina dough showed a relatively compact and continuous network structure, in which starch granules and Spirulina particles were more uniformly embedded. In contrast, when the addition level reached 4%, the gluten network appeared less complete, with visible holes and discontinuities.

At the weakening stage, the gluten network in the control dough became disordered after prolonged mixing. By comparison, doughs containing 1–3% Spirulina still exhibited a relatively continuous network structure. However, in the 4% Spirulina sample, the gluten structure became more fragmented and disordered, and the structural continuity was reduced.

**Figure 6:**
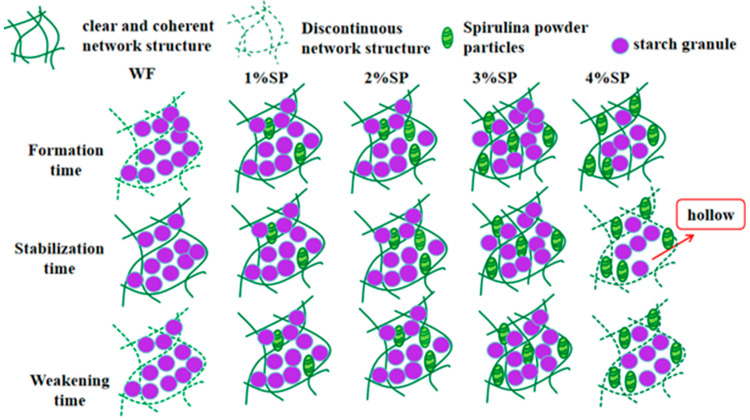
Schematic illustration of microstructure changes in doughs with different Spirulina additions during mixing.

Overall, moderate Spirulina addition was associated with a more continuous and integrated gluten network during mixing, whereas excessive addition was associated with a less complete network structure. These microstructural differences were generally consistent with the rheological behavior and water migration characteristics described above. Figure 6 presents a schematic diagram illustrating the proposed structural changes.

### 3.5. Effect of Spirulina Addition on Protein During Mixing

The formation and development of the gluten network during dough mixing are closely associated with protein polymerization, particularly intermolecular disulfide bond formation among gluten proteins [45]. Proteins were classified into three molecular weight regions: >60 kDa (high-molecular-weight region), 30–60 kDa (medium-molecular-weight region), and <30 kDa (low-molecular-weight region). In wheat dough, these regions typically contain high-molecular-weight glutenins, low-molecular-weight glutenins/gliadins, and low-molecular-weight proteins, respectively. However, because Spirulina contains endogenous proteins (primarily distributed around 20 kDa), the observed band patterns reflect contributions from both wheat and Spirulina proteins.

**Figure 7:**
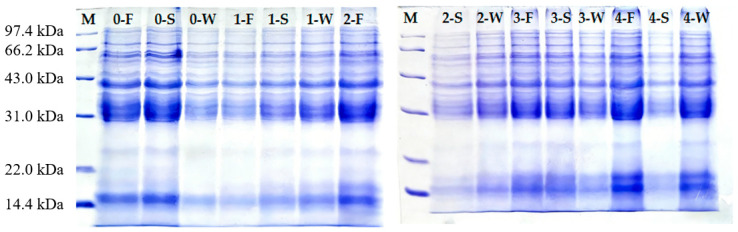
SDS-PAGE patterns of doughs with different Spirulina additions during mixing. M: low-molecular-weight protein marker; 0–4: Spirulina addition levels (%); F, S, and W represent development, stabilization, and weakening stages, respectively.

As shown in Figure 7 and Table 3, a new protein band appears at approximately 20 kDa following Spirulina addition. Given that spirulina proteins are predominantly distributed in the low-molecular-weight region (approximately 18 kDa) [46], changes in the intensity of this band reflect alterations in protein composition as well as potential shifts in disulfide bond-mediated aggregation states. While the ~20 kDa band is consistent with Spirulina proteins, contributions from Spirulina to other molecular weight regions cannot be excluded.

At the development stage, the >60 kDa fraction decreased and then slightly increased with Spirulina addition but remained lower than that of the control, suggesting a reduction in band intensity in the high-molecular-weight region. In contrast, the 30–60 kDa fraction increased and reached a maximum at 1% before declining, suggesting that low Spirulina addition may enhance dough viscoelasticity, whereas higher levels are unfavorable. At the stabilization stage, the >60 kDa fraction and its band intensity reached maximum values at 3% Spirulina addition, exceeding the control, suggesting an increase in band intensity in the high-molecular-weight region. This is consistent with the improved dough properties at this level, including higher stability and a more compact gluten network. At the weakening stage, the >60 kDa fraction decreased in Spirulina-containing samples, indicating reduced stability of high-molecular-weight aggregates during prolonged mixing. However, the 30–60 kDa fraction remained highest at 3%, suggesting that moderate Spirulina helped maintain protein fractions related to dough strength.

Considering both mixing stage and addition level, the 3% Spirulina sample showed increased band intensity in the >60 kDa region at the stabilization stage, suggesting that moderate addition is associated with enhanced gluten development. In contrast, at a higher addition level (4%), the intensity of high-molecular-weight aggregates decreased, which may be related to gluten dilution and changes in protein interactions. Overall, moderate Spirulina incorporation, particularly at 3%, was associated with greater band intensity in the high-molecular-weight region, which was consistent with the more developed gluten network observed by microscopy, whereas excessive addition appeared to interfere with these structural features. These observations are consistent with the improved dough strength, microstructure, and mixing resistance observed at moderate Spirulina levels, although the underlying molecular interactions require further investigation.

## 4. Conclusions

Spirulina incorporation significantly affected dough mixing behavior, rheology, water distribution, microstructure, and protein aggregation. Within the tested range, 3% Spirulina addition exhibited the best performance, improving development time and stability time while reducing the degree of softening. Compared with the 3% inclusion level, the 4% Spirulina addition exhibited less favorable performance, including reduced stability and increased weakening. The LF-NMR results showed that A_21_ exhibited an increasing trend (*p* > 0.05), accompanied by a prolongation of T_22_, indicating enhanced water mobility and reduced binding strength. Among all treatments, ~3% Spirulina provided the optimal balance between structural reinforcement and gluten dilution, resulting in improved dough stability and mixing tolerance. These findings provide a mechanistic basis for the application of Spirulina in cereal systems. The present study evaluated multiple properties of the dough but did not focus on any specific wheat flour-based food product. The fundamental findings of this work can serve as a basis for future investigations into the specific applications of Spirulina-containing doughs in various food products.

## Figures and Tables

**Table 1:** Farinograph and mixograph parameters of wheat flour and spirulina blends.

Sample	WF	1% SP	2% SP	3% SP	4% SP
Water absorption (%)	56.2 ± 0.90 a	58.8 ± 1.00 a	59.6 ± 1.10 a	58.86 ± 1.00 a	60.4 ± 1.20 a
Formation time (min)	2.2 ± 0.20 ab	2.7 ± 0.30 ab	2.4 ± 0.20 ab	2.6 ± 0.30 a	1.9 ± 0.20 b
Stabilization time (min)	4.9 ± 0.40 cd	4.8 ± 0.40 d	5.2 ± 0.40 bc	6.4 ± 0.50 a	5.6 ± 0.40 b
Degree of weakening (FE)	98 ± 4.00 a	88 ± 3.50 b	76 ± 3.00 c	63 ± 2.50 e	67 ± 2.80 d
Powder quality index (mm)	59 ± 2.50 cd	60 ± 2.50 d	66 ± 2.80 bc	81 ± 3.50 a	70 ± 3.00 b
Peak time (min)	2.46 ± 0.13 c	2.94 ± 0.09 bc	3.06 ± 0.08 abc	3.16 ± 0.22 ab	3.14 ± 0.11 ab
8 min bandwidth (%)	14.33 ± 2.78 a	9.76 ± 0.03 b	9.53 ± 0.21 b	10.03 ± 0.47 b	10.20 ± 0.75 b
Peak curve area (% TQ min)	115.64 ± 0.64 b	138.89 ± 2.00 b	143.23 ± 2.99 a	145.06 ± 6.28 a	145.27 ± 4.43 a
Right Slope	1.80 ± 0.53 a	3.71 ± 0.01 b	3.69 ± 0.06 b	3.27 ± 0.05 ab	3.05 ± 0.93 ab

Data are expressed as mean ± standard deviation. Different superscript letters within the same row indicate significant differences (*p* < 0.05).

**Table 2:** Changes in moisture states of doughs with different Spirulina additions during mixing.

Samples	Sampling	T_21_	T_22_	T_23_	A_21_	A_22_	A_23_
WF	formation stage	0.31 ± 0.02 ^Ab^	52.28 ± 1.86 ^Aa^	248.24 ± 24.81 ^Aa^	8.36 ± 0.33 ^Aa^	91.13 ± 0.35 ^Aa^	0.51 ± 0.03 ^Ca^
	stabilization stage	0.34 ± 0.00 ^Aa^	50.44 ± 0.07 ^Aab^	200.65 ± 6.19 ^ABb^	9.1 ± 0.98 ^Aa^	90.22 ± 0.99 ^Aa^	0.68 ± 0.01 ^Ba^
	weakening stage	0.29 ± 0.02 ^Ac^	50.49 ± 0.02 ^Aa^	161.77 ± 19.65 ^Bb^	8.66 ± 0.31 ^Aa^	90.53 ± 0.32 ^Aa^	0.81 ± 0.01 ^Aa^
1% SP	formation stage	0.33 ± 0.04 ^Ab^	50.51 ± 0.00 ^Aa^	260.22 ± 12.84 ^Aa^	9.92 ± 0.6 ^Aa^	89.59 ± 0.59 ^Aa^	0.49 ± 0.01 ^Aa^
	stabilization stage	0.42 ± 0.03 ^Aa^	52.34 ± 1.96 ^Aa^	256.27 ± 8.89 ^Ab^	9.56 ± 0.70 ^Aa^	89.93 ± 0.75 ^Aa^	0.51 ± 0.06 ^Ab^
	weakening stage	0.35 ± 0.04 ^Abc^	50.6 ± 0.09 ^Aa^	196.92 ± 3.95 ^Bab^	9.12 ± 2.22 ^Aa^	90.24 ± 2.26 ^Aa^	0.64 ± 0.04 ^Ab^
2% SP	formation stage	0.42 ± 0.00 ^Aab^	50.37 ± 0.05 ^Aa^	256.55 ± 48.11 ^Aa^	9.62 ± 0.63 ^Aa^	90.01 ± 0.59 ^Aa^	0.38 ± 0.04 ^Bb^
	stabilization stage	0.44 ± 0.08 ^Aa^	52.39 ± 1.84 ^Aa^	261.02 ± 25.60 ^ABa^	9.05 ± 1.14 ^Aa^	90.58 ± 1.16 ^Aa^	0.37 ± 0.01 ^Bc^
	weakening stage	0.36 ± 0.03 ^Abc^	48.76 ± 1.71 ^Aa^	195.28 ± 20.26 ^Bab^	10.38 ± 0.10 ^Aa^	89.01 ± 0.12 ^Aa^	0.61 ± 0.02 ^Abc^
3% SP	formation stage	0.4 ± 0.04 ^Aab^	48.88 ± 1.68 ^Aa^	220.29 ± 27.09 ^Aa^	9.44 ± 0.06 ^Aa^	90.1 ± 0.02 ^Aa^	0.47 ± 0.04 ^Aab^
	stabilization stage	0.42 ± 0.09 ^Aa^	50.53 ± 0.02 ^Aab^	235.41 ± 11.98 ^Ab^	9.32 ± 0.17 ^Aa^	90.22 ± 0.16 ^Aa^	0.46 ± 0.01 ^Abc^
	weakening stage	0.48 ± 0.03 ^Aa^	48.88 ± 1.68 ^Aa^	240.38 ± 25.07 ^Aa^	9.33 ± 0.23 ^Aa^	90.2 ± 0.21 ^Aa^	0.48 ± 0.02 ^Ac^
4% SP	formation stage	0.45 ± 0.03 ^Aa^	48.84 ± 1.68 ^Aa^	204.77 ± 3.68 ^Aa^	9.2 ± 0.35 ^Aa^	90.28 ± 0.34 ^Aa^	0.52 ± 0.01 ^Aa^
	stabilization stage	0.34 ± 0.02 ^Aa^	46.96 ± 0.13 ^Ab^	205 ± 10.54 ^Ab^	9.08 ± 0.59 ^Aa^	90.43 ± 0.64 ^Aa^	0.49 ± 0.05 ^Abc^
	weakening stage	0.42 ± 0.03 ^Aab^	47.21 ± 0.02 ^Aa^	216.98 ± 22.51 ^Aab^	8.98 ± 0.02 ^Aa^	90.52 ± 0.09 ^Aa^	0.5 ± 0.06 ^Ac^

**Notes:** Different superscript letters within the same column indicate significant differences. Uppercase letters indicate significant differences among mixing stages, while lowercase letters indicate significant differences among Spirulina addition levels (*p* < 0.05).

**Table 3:** Changes in protein molecular weight distribution of doughs with different Spirulina additions during mixing.

Samples	Mixing Stage	Protein Proportion (%)		
		>60 kDa	30–60 kDa	<30 kDa
WF	formation stage	24.752 ± 1.20 ^a^	67.204 ± 3.20 ^b^	8.043 ± 0.65 ^c^
	stabilization stage	17.646 ± 1.10 ^b^	74.120 ± 3.50 ^a^	8.236 ± 0.70 ^c^
	weakening stage	22.739 ± 1.40 ^a^	71.293 ± 2.00 ^b^	5.967 ± 0.45 ^b^
1% SP	formation stage	20.737 ± 1.10 ^b^	72.279 ± 3.50 ^a^	6.987 ± 0.60 ^c^
	stabilization stage	14.348 ± 0.90 ^c^	48.974 ± 2.80 ^c^	36.678 ± 2.50 ^a^
	weakening stage	18.857 ± 1.10 ^b^	75.530 ± 1.80 ^a^	5.613 ± 0.40 ^b^
2% SP	formation stage	21.427 ± 1.05 ^b^	70.451 ± 3.40 ^a^	8.122 ± 0.70 ^c^
	stabilization stage	16.793 ± 1.00 ^b^	73.272 ± 3.40 ^a^	9.935 ± 0.80 ^c^
	weakening stage	17.134 ± 1.00 ^bc^	76.150 ± 1.90 ^a^	6.714 ± 0.50 ^b^
3% SP	formation stage	16.529 ± 0.90 ^c^	65.568 ± 3.10 ^b^	17.903 ± 1.20 ^b^
	stabilization stage	22.932 ± 1.40 ^a^	60.133 ± 3.00 ^b^	16.935 ± 1.20 ^b^
	weakening stage	18.441 ± 1.05 ^b^	76.505 ± 1.85 ^a^	5.053 ± 0.35 ^b^
4% SP	formation stage	21.575 ± 1.08 ^b^	52.734 ± 2.80 ^c^	25.691 ± 1.80 ^a^
	stabilization stage	15.784 ± 0.95 ^bc^	72.052 ± 3.30 ^a^	12.165 ± 0.90 ^bc^
	weakening stage	16.310 ± 0.95 ^c^	69.266 ± 2.10 ^b^	14.424 ± 1.00 ^a^

**Notes:** Values are presented as mean ± SD (*n* = 3). Different superscript letters (a, b, c) within the same row indicate significant differences among Spirulina addition levels at *p* < 0.05 (one-way ANOVA followed by Tukey’s HSD post hoc test).

## Data Availability

The original contributions presented in this study are included in the article. Further inquiries can be directed to the corresponding author.
